# Random Survival Forests to Predict Disease Control for Hepatocellular Carcinoma Treated With Transarterial Chemoembolization Combined With Sorafenib

**DOI:** 10.3389/fmolb.2021.618050

**Published:** 2021-05-20

**Authors:** Bin-Yan Zhong, Zhi-Ping Yan, Jun-Hui Sun, Lei Zhang, Zhong-Heng Hou, Xiao-Li Zhu, Ling Wen, Cai-Fang Ni

**Affiliations:** ^1^ Department of Interventional Radiology, The First Affiliated Hospital of Soochow University, Suzhou, China; ^2^ Department of Interventional Radiology, Zhongshan Hospital, Fudan University, Shanghai, China; ^3^ Shanghai Institution of Medical Imaging, Shanghai, China; ^4^ National Clinical Research Center for Interventional Medicine, Shanghai, China; ^5^ Hepatobiliary and Pancreatic Interventional Treatment Center, Division of Hepatobiliary and Pancreatic Surgery, The First Affiliated Hospital, Zhejiang University School of Medicine, Hangzhou, China; ^6^ Department of Radiology, The First Affiliated Hospital of Soochow University, Suzhou, China

**Keywords:** hepatocellular carcinoma, transarterial chemoembolization, sorafenib, disease control, random survival forest

## Abstract

**Objectives:** To use baseline variables to predict one-year disease control for patients with hepatocellular carcinoma (HCC) treated with transarterial chemoembolization (TACE) combined with sorafenib as initial treatment by applying a machine learning approach based on the random survival forest (RF) model.

**Materials and Methods:** The multicenter retrospective study included 496 patients with HCC treated with TACE combined with sorafenib between January 2014 and December 2018. The independent risk factors associated with one-year disease control (complete response, partial response, stable disease) were identified using the RF model, and their predictive importance was determined using the Gini index. Tumor response was assessed according to modified Response Evaluation Criteria in Solid Tumors.

**Results:** The median overall survival was 15.5 months. A total of 186 (37.5%) patients achieved positive one-year disease control. The Barcelona Clinic Liver *Cancer* (BCLC) stage (Gini index: 20.0), tumor size (≤7 cm, >7 cm; Gini index: 9.0), number of lobes involved (unilobar, bilobar; Gini index: 6.4), alpha-fetoprotein level (≤200 ng/dl, >200 ng/dl; Gini index: 6.1), albumin–bilirubin grade (Gini index: 5.7), and number of lesions (1, >1; Gini index: 5.3) were identified as independent risk factors, with the BCLC stage as the most important variable. The RF model achieved a higher concordance index of 0.724 compared to that for the logistic regression model (0.709).

**Conclusions:** The RF model is a simple and accurate approach for prediction of one-year disease control for patients with HCC treated with TACE combined with sorafenib.

## Introduction

Despite improving surveillance programs, around 80% of hepatocellular carcinomas (HCCs) are first diagnosed at an intermediate or advanced stage according to the Barcelona Clinic Liver *Cancer* (BCLC) staging system (Bray et al., 2018; Forner et al., 2018; Villanueva, 2019). For intermediate-stage HCC, transarterial chemoembolization (TACE) is the standard approach recommended by the American Association for the Study of the Liver Disease (AASLD) and European Association for the Study of the Liver (EASL) guidelines (European Association for the Study of the Liver, 2018; Marrero et al., 2018). According to the BRIDGE study, TACE is the most widely applied method for both intermediate and advanced HCCs in real-world clinical practice (Park et al., 2015). Nevertheless, the prognosis of patients treated with TACE varies from a median survival of 19.4 months generally to around 49.1 months in well-selected patients, which is mainly due to the high heterogeneity of unresectable HCC (Lencioni et al., 2016a; Galle et al., 2017).

Due to the fact that there is an increase in vascular endothelial growth factor after TACE, the combination of TACE with sorafenib, an orally active multikinase inhibitor with antiangiogenic properties, should improve the efficacy of TACE ideally (Li et al., 2004; Wang et al., 2008). Unfortunately, three randomized controlled trials (RCTs) failed to identify significant treatment efficacy and safety for TACE combined with sorafenib compared to TACE monotherapy (Kudo et al., 2011; Lencioni et al., 2016b; Meyer et al., 2017). On the contrary, a recently reported RCT carried out by Kudo et al., the TACTICS trial, demonstrated positive results (Kudo et al., 2019). Notably, a much longer median duration of sorafenib administration was observed in the TACTICS trial compared to that in the previous three negative trials, which might be a key reason for the success of the TACTICS trial (Kudo et al., 2019). Therefore, a longer time of disease control in order to achieve a longer sorafenib administration period is an important factor for patients achieving survival benefit from the combination treatment of TACE and sorafenib (Kudo and Arizumi, 2017).

As mentioned before, high heterogeneity of unresectable HCC leads to the diverse prognosis including the sorafenib administration period for patients treated with TACE combined with sorafenib. The prognosis of HCC is mainly based on tumor burden and liver function. Recently, a machine learning approach, random survival forest (RF), has been applied as an intuitive technique for predicting individual prognosis (Hsich et al., 2011; Hu and Steingrimsson, 2018). It requires little input from the analyst and has ability to easily deal with nonlinear effects and variable interactions, which are major limitations of conventional linear discriminant analysis (Ishwaran et al., 2014). By combining many individual decision trees, RFs form an ensemble method and provide an accurate assessment of variable importance of every individual variable associated with prognosis (Hu and Steingrimsson, 2018).

The present study aimed to predict one-year disease control for unresectable HCC treated with TACE combined with sorafenib by applying an RF model. In addition, the study also evaluated the importance and predictive value of variables in the RF model for a one-year disease control outcome.

## Materials and Methods

### Patients’ Criteria

This multicenter retrospective study included patients diagnosed with unresectable HCC according to the AASLD/EASL guidelines and treated with TACE combined with sorafenib as initial treatment between January 2014 and December 2018 at three institutions. The study was approved by the institutional review boards at the three institutions, and the requirement for informed consent was waived due to its retrospective nature. The study was performed in accordance with the Declaration of Helsinki. The inclusion criteria were as follows: 1) 18 years or older with the definite diagnosis of HCC; 2) having an Eastern Cooperative Oncology Group performance score of 0 or 1; 3) not suitable or unwilling to receive curative treatment such as resection, ablation, or transplantation; and 4) no prior HCC-related treatment. Patients were excluded if they had any of the following: 1) Child–Pugh grade C or aspartate transaminase >5 times the upper limit of the normal range and total bilirubin >1.5 times the upper limit of the normal range; 2) inadequate renal, clotting, and hematologic function; 3) accompanying or history of any other primary malignancies; and 4) incomplete or missing clinical and follow-up data. Multidisciplinary discussion was carried out before treatment to determine if TACE combined with sorafenib was the recommended therapy for the patients. Written informed consent regarding the advantages and disadvantages of the combination treatment, including the potential treatment outcomes, treatment-related morbidities, and costs, was obtained from every included patient.

### Treatment

Patients included in the study received the conventional TACE procedure, and details on it have been provided in our previous studies (Zhong et al., 2017). Repeat TACE was assessed and provided according to the “on demand” mode: subsequent contrast-enhanced computed tomography (CT) or magnetic resonance imaging (MRI) follow-up was carried out 4–6 weeks after the previous procedure. TACE was discontinued when no vital active tumor lesion(s) was observed during follow-up CT/MRI, and the patient underwent the next contrast-enhanced CT/MRI plus alpha-fetoprotein follow-up every 8–10 weeks. Repeat TACE was evaluated if the contrast-enhanced CT/MRI presented new lesions (Terzi et al., 2012).

Sorafenib (Bayer Healthcare, Leverkusen, Germany) was administered within 3–7 days after every TACE with an initial dose of 400 mg twice daily. It was temporary stopped the day before every TACE. Dose reductions to 200 mg twice daily or 200 mg once daily or temporary interruptions were allowed due to drug-related toxicity. Sorafenib was discontinued in the event of disease progression or unacceptable toxicity.

The primary outcome of the study was one-year disease control, defining patients achieving complete response (CR), partial response (PR), or stable disease (SD) according to modified Response Evaluation Criteria in Solid Tumors (mRECIST) with a period no less than 1 year after initial TACE. Tumor response was assessed by two independent radiologists (__ and __) with more than 5 years of experience in diagnostic radiology through the PACS (NEUSOFTPACS/RIS, Shengyang Neusoft Co., Ltd., China). A third radiologist (___) made the final decision in case of disagreement.

### Establishment of the RF Model

Variables identified as independently associated with the primary outcome by univariate and multivariate logistic analyses were introduced to establish the RF model. All data were randomly divided into a training set and a validation set with a 5:3 ratio. The RF model is trained by growing a large number of individual trees, and each tree is trained on a random-bootstrap sample from the original cohort (Hsich et al., 2011; Hu and Steingrimsson, 2018). Details on the theory of how the RF model was established have been reported previously (Ingrisch et al., 2018).

### Statistical Analysis

Categorical variables are presented as frequencies and percentages, and continuous variables are presented as medians with 95% confidence intervals (CIs) or means with standard deviations. Variables with a *P* value no more than 0.20 in the univariate logistic analysis were considered strong risk factors associated with the primary outcome and were put into the multivariate logistic analysis. Variables with *P* values no more than 0.05 were considered independent risk factors associated with the primary outcome. The RF model was established based on the independent risk factors. The predictive performance of the RF model and the traditional logistic model was validated internally using the concordance c statistic (C-index). The Gini index was applied to describe the importance of the variables in the RF model associated with the primary outcome (Jain et al., 2018). Statistical analyses were performed using SPSS version 22.0 software for Windows (IBM Corporation, Somers, NY, United States), and the RF model was established in the R package “randomForest” (https://www.stat.berkeley.edu/∼breiman/RandomForests/).

## Results

### Patient Characteristics

**FIGURE 1:**
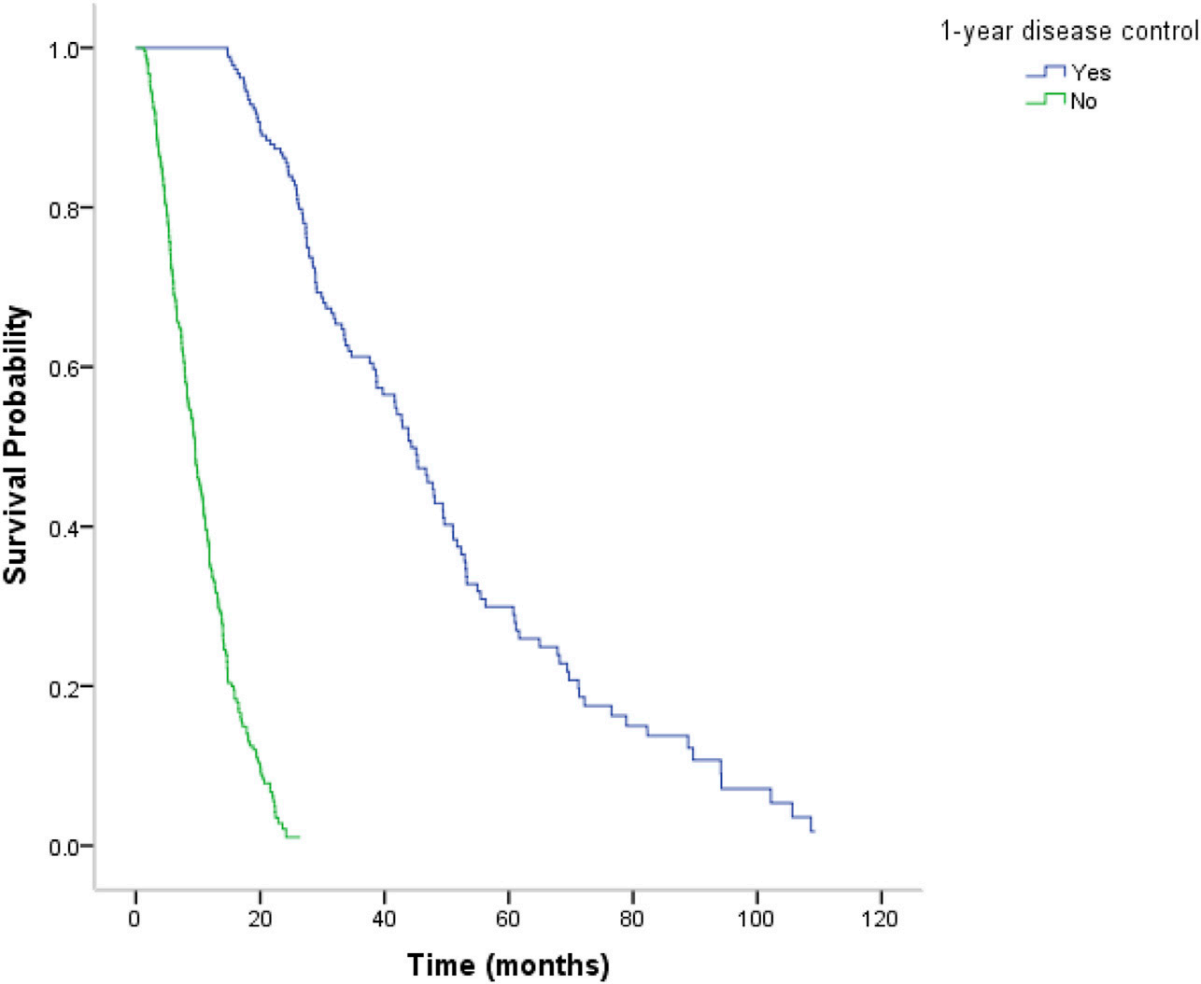
Kaplan–Meier analysis of median overall survival (OS). The median OS was 44.3 months for patients with positive one-year disease control (CR/PR/SD) and was significantly longer for patients with negative one-year disease control (progression disease) (9.5 months) (*P* < 0.001).

The study included 496 patients (427 males, 69 females; mean age, 54 years; range, 21–81 years), with 313, 59, and 124 patients from institutions A, B, and C, respectively. The baseline characteristics of included patients are presented in Table 1. There were 186 (37.5%) patients who achieved CR/PR/SD at least 1 year after initial treatment. The median overall survival (OS) was 15.5 months, with that of 14.8, 25.9, and 14.8 (*p* = 0.142) months in institutions A, B, and C, respectively. The median OS was significantly longer for patients with positive one-year disease control (CR/PR/SD) compared to that of patients with negative one-year disease control (progression disease) (44.3 months vs. 9.5 months; *p* < 0.001) (Figure 1). No TACE or sorafenib treatment–related death occurred.

**TABLE 1:** Patient characteristics.

Characteristic	Overall (*n* = 496)	Institution A (*n* = 313)		Institution B (*n* = 59)	Institution C (*n* = 124)	*p* ^*^
Gender						0.196
Male	427 (86.1%)	269 (85.9%)		47 (79.7%)	111 (89.5%)	
Female	69 (13.9%)	44 (14.1%)		12 (20.3%)	13 (10.5%)	
Hepatitis B						0.428
Yes	417 (84.1%)	268 (85.6%)		49 (83.1%)	100 (80.6%)	
No	79 (15.9%)	45 (14.4%)		10 (16.9%)	24 (19.4%)	
Age (y)						0.019
≤55	321 (64.7%)	217 (69.3%)		33 (55.9%)	71 (57.3%)	
>55	175 (35.3%)	96 (30.7%)		26 (44.1%)	53 (42.7%)	
ALBI grade						0.523
1	251 (50.6%)	163 (52.1%)		26 (44.1%)	62 (50.0%)	
2	245 (49.4%)	150 (47.9%)		33 (55.9%)	62 (50.0%)	
Child–Pugh grade						0.430
A	430 (86.7%)	273 (87.2%)		48 (81.4%)	109 (87.9%)	
B	66 (13.3%)	40 (12.8%)		11 (18.6%)	15 (12.1%)	
Maximum tumor size (cm)						0.417
<5	194 (39.1%)	119 (38.0%)		27 (45.8%)	48 (38.7%)	
5–10	180 (36.3%)	118 (37.7%)		22 (37.3%)	40 (32.3%)	
>10	122 (24.6%)	76 (24.3%)		10 (16.9%)	36 (29.0%)	
Lobes involved						0.852
Unilobar	318 (64.1%)	198 (63.3%)		38 (64.4%)	82 (66.1%)	
Bilobar	178 (35.9%)	115 (36.7%)		21 (35.6%)	42 (33.9%)	
No. of lesions						0.779
1	225 (45.4%)	144 (46.0%)		28 (47.5%)	53 (42.7%)	
>1	271 (54.6%)	169 (54.0%)		31 (52.5%)	71 (57.3%)	
ECOG						0.090
0	482 (97.2%)	307 (98.1%)		58 (98.3%)	117 (94.4%)	
1	14 (2.8%)	6 (1.9%)		1 (1.7%)	7 (5.6%)	
PVTT						0.005
None	309 (62.3%)	194 (62.0%)		44 (74.6%)	71 (57.3%)	
Branch	102 (20.6%)	71 (22.7%)		11 (18.6%)	20 (16.1%)	
Main	85 (17.1%)	48 (15.3%)		4 (6.8%)	33 (26.6%)	
BCLC stage						0.071
B	252 (50.8%)	156 (49.8%)		38 (64.4%)	58 (46.8%)	
C	244 (49.2%)	157 (50.2%)		21 (35.6%)	66 (53.2%)	
AFP (ng/dl)						0.760
≤200	257 (51.8%)	159 (50.8%)		33 (55.9%)	65 (52.4%)	
>200	239 (48.2%)	154 (49.2%)		26 (44.1%)	59 (47.6%)	
AST (U/L)						0.138	
≤40	218 (44.0%)	129 (41.2%)		25 (42.4%)	64 (51.6%)	
>40	278 (56.0%)	184 (58.8%)		34 (57.6%)	60 (48.4%)	
ALT (U/L)						0.134	
≤40	284 (57.3%)	174 (55.6%)		30 (50.8%)	80 (64.5%)	
>40	212 (42.7%)	139 (44.4%)		29 (49.2%)	44 (35.5%)	

* The chi-square test was used. ALBI, albumin–bilirubin; ECOG, Eastern Cooperative Oncology Group; PVTT, portal vein tumor thrombosis; BCLC, Barcelona Clinic Liver *Cancer*; AFP, alpha-fetoprotein; AST, aspartate transaminase; ALT, alanine transaminase.

### Strong and Independent Risk Factors Associated With One-Year Disease Control

After univariate logistic analysis using potentially significant variables, seven variables including the BCLC stage, tumor size (≤7 cm, >7 cm), alpha-fetoprotein level (≤200 ng/dl, >200 ng/dl), albumin–bilirubin (ALBI) grade, number of lesions (1, >1), number of lobes involved (unilobar, bilobar), and age (≤55 years, >55 years) were identified as strong risk factors associated with one-year disease control (Table 2). Multivariate logistic analysis using these seven strong risk factors was then performed, and six variables including the BCLC stage, tumor size (≤7 cm, >7 cm), alpha-fetoprotein level (≤200 ng/dl, >200 ng/dl), ALBI grade, number of lesions (1, >1), and number of lobes involved (unilobar, bilobar) were finally identified as independent risk factors associated with one-year disease control (Table 3).

**TABLE 2:** Univariate analysis of risk factors associated with one-year disease control.

Variables	HR	95% CI	B-values*	*p* value**
BCLC stage				<0.001
B	1			
C	5.712	3.590–9.087	1.742	
Maximum tumor size (cm)				<0.001
≤7	1			
>7	3.485	2.346–5.177	1.248	
AFP (ng/ml)				0.001
≤200	1			
>200	2.086	1.211–4.237	0.735	
ALBI grade				0.002
1	1			
2	2.024	1.296–3.162	0.705	
No. of lesions				0.003
1	1			
>1	1.744	1.208–2.518	0.556	
Lobes involved				0.006
Unilobar	1			
Bilobar	2.038	1.223–3.396	0.712	
Age (y)				0.110
≤55	1			
>55	0.689	0.437–1.089	−0.372	

* B-values are regression coefficients.

** Univariate logistic regression analysis was used. BCLC, Barcelona Clinic Liver *Cancer*; AFP, alpha-fetoprotein; ALBI, albumin–bilirubin.

**TABLE 3:** Multivariate analysis of risk factors associated with one-year disease control.

Variables	HR	95% CI	B-values*	*p* value**
BCLC stage				
B	1			
C	5.657	3.544–9.029	1.733	<0.001
Maximum tumor size (cm)				
≤7	1			
>7	2.387	1.491–3.821	0.870	<0.001
AFP (ng/ml)				
≤200	1			
>200	2.106	1.345–3.297	0.745	0.001
ALBI grade				
1	1			
2	1.906	1.225–2.966	0.645	0.004
No. of lesions				
1	1			
>1	2.218	1.320–3.728	0.797	0.003
Lobes involved				
Unilobar	1			
Bilobar	1.786	1.059–3.014	0.580	0.030

* B-values are regression coefficients.

** Multivariate logistic regression analysis was used. BCLC, Barcelona Clinic Liver *Cancer*; AFP, alpha-fetoprotein; ALBI, albumin–bilirubin.

### Establishment of the RF Model and Importance of the Variables in the RF Model

**FIGURE 2:**
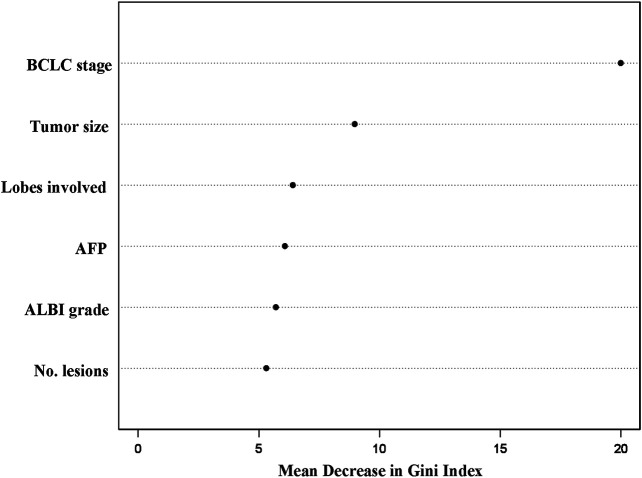
Order of importance of the variables in the random survival forest model for one-year disease prediction. BCLC, Barcelona Clinic Liver *Cancer*; AFP, alpha-fetoprotein; ALBI, albumin–bilirubin.

The RF model was established based on the identified independent risk factors (Figure 2). The predictive performance of the trained RF model was better than that of the traditional logistic model, with the C-indexes of 0.724 and 0.709, respectively.

The importance of the variables in the RF model is illustrated in Figure 2. The BCLC stage showed the highest Gini index (20.0), following tumor size (≤7 cm, >7 cm; Gini index: 9.0), number of lobes involved (unilobar, bilobar; Gini index: 6.4), alpha-fetoprotein level (≤200 ng/dl, >200 ng/dl; Gini index: 6.1), ALBI grade (Gini index: 5.7), and number of lesions (1, >1; Gini index: 5.3).

## Discussion

By applying a machine learning approach, the random survival forest model, the present study demonstrated that the BCLC stage, tumor size, alpha-fetoprotein level, ALBI grade, number of lesions, and number of lobes involved were independent risk factors associated with one-year disease control for unresectable HCC treated with TACE combined with sorafenib. The importance and predictive value of these independent risk factors were assessed and ranked based on the Gini index, with the BCLC stage and number of lesions showing highest and lowest importance and predictive values, respectively. According to the C-index, the predictive performance of the RF model was better than that of the traditional logistic model.

The study identified that the BCLC stage had highest importance and predictive value associated with one-year disease control. Combining TACE with sorafenib, which is the standard recommendation for advanced HCC, should achieve a synergetic effect ideally. Nevertheless, no randomized controlled trial (RCT) has been provided with positive results of this combination therapy for advanced HCC (Park et al., 2019). The only RCT comparing TACE combined with sorafenib vs. sorafenib monotherapy for advanced HCC, the STAH trial, demonstrated that there was no significant survival difference between TACE combined with sorafenib and sorafenib monotherapy for advanced HCC (median OS: 12.8 months vs. 10.8 months; *p* = 0.290) (Park et al., 2019). A relatively short period of sorafenib administration was observed (166 days) for advanced HCC treated with TACE combined with sorafenib in this trial.

Patients with HCC are heterogeneous regarding tumor burden, and previous studies have identified tumor burden as a robust risk factor associated not only with TACE monotherapy but also with TACE combined with sorafenib (Wang et al., 2019). Radiological response rates decrease as the tumor burden increases for patients treated with TACE (Kim et al., 2015). The present study demonstrated that tumor burden including the tumor size, number of lesions, and number of lobes involved were independent risk factors associated with the radiological response rate (one-year disease control) for patients treated with TACE combined with sorafenib.

This study applied the ALBI grade to assess the association between pre-treatment liver function and one-year disease control. The ALBI grade is based solely on two objective variables, which are serum albumin and bilirubin. The ALBI grade was first introduced by Johnson and colleagues in 2015, and it was then identified that its prognostic performance was at least no worse than that of the Child–Pugh grade for patients with HCC treated with various treatments (Johnson et al., 2015). Considering the objectivity and easy application, the ALBI grade is recommended as an alternative method for liver function assessment for HCC (Hiraoka et al., 2019). Patients with unresectable HCC are heterogeneous regarding liver function (Bolondi et al., 2012; Weinmann et al., 2015). The sorafenib administration period is shortened if deterioration of liver function occurs, even though a global real-world study demonstrated that sorafenib is safe and effective for HCC with different liver functions (Marrero et al., 2016). The present study demonstrated that low ALBI grade was an indicator of longer disease control for patients with unresectable HCC treated with TACE combined with sorafenib.

This study has several limitations. First, the retrospective nature of the study might cause selection bias of the included patients. Nevertheless, no significant difference regarding the baseline characteristics except for the age of the included patients between the three institutions was observed. Second, the median OS in institution B was much longer than that in institutions A and C. It might be mainly due to the relatively lower tumor burden of the patients in institution A compared to that in the other two institutions. Third, this study did not analyze independent risk factors associated with longer disease control such as two-year disease control. Fourth, due to the incomplete data, we were unable to collect and analyze the association between dose reduction of sorafenib and treatment outcome. Further work is encouraged to explore the association between dose reduction and prognosis for HCC treated with TACE combined with sorafenib. Fifth, due to the lack of the external validation cohort, the accuracy of the random survival model was just validated internally. Further work should be carried out to validate the accuracy of the random survival model in an independent external cohort. Finally, the study only included patients treated with TACE combined with sorafenib. It is better to include a control group for patients treated with TACE monotherapy to identify the optimal candidates to achieve longer disease control for unresectable HCC treated with TACE combined with sorafenib.

In conclusion, by applying a machine learning approach, the present study establishes a random survival forest model including the BCLC stage, tumor size, alpha-fetoprotein level, ALBI grade, number of lesions, and number of lobes involved to accurately predict one-year disease control for unresectable HCC treated with TACE combined with sorafenib. The predictive performance of the random survival forest model is better than that of the traditional logistic model.

## Data Availability

The original contributions presented in the study are included in the article/Supplementary Material, and further inquiries can be directed to the corresponding authors.

## References

[B1] Bolondi L. Burroughs A. Dufour J. F. Galle P. R. Mazzaferro V. Piscaglia F. (2012). Heterogeneity of Patients with Intermediate (BCLC B) Hepatocellular Carcinoma: Proposal for a Subclassification to Facilitate Treatment Decisions. Semin. Liver Dis. 32 (4), 348–359. 10.1055/s-0032-1329906 23397536

[B2] Bray F. Ferlay J. Soerjomataram I. Siegel R. L. Torre L. A. Jemal A. (2018). Global Cancer Statistics 2018: GLOBOCAN Estimates of Incidence and Mortality Worldwide for 36 Cancers in 185 Countries. CA: A Cancer J. Clinicians 68 (6), 394–424. 10.3322/caac.21492 30207593

[B3] European Association for the Study of the Liver (2018). Electronic address, e.e.e., and European Association for the Study of the, L. (EASL Clinical Practice Guidelines: Management of hepatocellular carcinoma. J. Hepatol. 69 (1), 182–236. 10.1016/j.jhep.2018.03.019 29628281

[B4] Forner A. Reig M. Bruix J. (2018). Hepatocellular Carcinoma. Lancet 391 (10127), 1301–1314. 10.1016/S0140-6736(18)30010-2 29307467

[B5] Galle P. R. Tovoli F. Foerster F. Wörns M. A. Cucchetti A. Bolondi L. (2017). The Treatment of Intermediate Stage Tumours beyond TACE: From Surgery to Systemic Therapy. J. Hepatol. 67 (1), 173–183. 10.1016/j.jhep.2017.03.007 28323121

[B6] Hiraoka A. Kumada T. Michitaka K. Kudo M. (2019). Newly Proposed ALBI Grade and ALBI-T Score as Tools for Assessment of Hepatic Function and Prognosis in Hepatocellular Carcinoma Patients. Liver Cancer 8 (5), 312–325. 10.1159/000494844 31768342 PMC6873026

[B7] Hsich E. Gorodeski E. Z. Blackstone E. H. Ishwaran H. Lauer M. S. (2011). Identifying Important Risk Factors for Survival in Patient with Systolic Heart Failure Using Random Survival Forests. Circ. Cardiovasc. Qual. Outcomes 4 (1), 39–45. 10.1161/CIRCOUTCOMES.110.939371 21098782 PMC3991475

[B8] Hu C. Steingrimsson J. A. (2018). Personalized Risk Prediction in Clinical Oncology Research: Applications and Practical Issues Using Survival Trees and Random Forests. J. Biopharm. Stat. 28 (2), 333–349. 10.1080/10543406.2017.1377730 29048993 PMC7196339

[B9] Ingrisch M. Schöppe F. Paprottka K. Fabritius M. Strobl F. F. De Toni E. N. (2018). Prediction of 90Y Radioembolization Outcome from Pretherapeutic Factors with Random Survival Forests. J. Nucl. Med. 59 (5), 769–773. 10.2967/jnumed.117.200758 29146692

[B10] Ishwaran H. Gerds T. A. Kogalur U. B. Moore R. D. Gange S. J. Lau B. M. (2014). Random Survival Forests for Competing Risks. Biostatistics 15 (4), 757–773. 10.1093/biostatistics/kxu010 24728979 PMC4173102

[B11] Jain S. S. Sarkar I. N. Stey P. C. Anand R. S. Biron D. R. Chen E. S. (2018). Using Demographic Factors and Comorbidities to Develop a Predictive Model for ICU Mortality in Patients with Acute Exacerbation COPD. AMIA Annu. Symp. Proc. 2018, 1319–1328. 30815176 PMC6371239

[B12] Johnson P. J. Berhane S. Kagebayashi C. Satomura S. Teng M. Reeves H. L. (2015). Assessment of Liver Function in Patients with Hepatocellular Carcinoma: a New Evidence-Based Approach-The ALBI Grade. Jco 33 (6), 550–558. 10.1200/JCO.2014.57.9151 PMC4322258 25512453

[B13] Kim B. K. Kim S. U. Kim K. A. Chung Y. E. Kim M.-J. Park M.-S. (2015). Complete Response at First Chemoembolization Is Still the Most Robust Predictor for Favorable Outcome in Hepatocellular Carcinoma. J. Hepatol. 62 (6), 1304–1310. 10.1016/j.jhep.2015.01.022 25637785

[B14] Kudo M. Arizumi T. (2017). Transarterial Chemoembolization in Combination with a Molecular Targeted Agent: Lessons Learned from Negative Trials (Post-TACE, BRISK-TA, SPACE, ORIENTAL, and TACE-2). Oncology 93 (Suppl. 1), 127–134. 10.1159/000481243 29258086

[B15] Kudo M. Imanaka K. Chida N. Nakachi K. Tak W.-Y. Takayama T. (2011). Phase III Study of Sorafenib after Transarterial Chemoembolisation in Japanese and Korean Patients with Unresectable Hepatocellular Carcinoma. Eur. J. Cancer 47 (14), 2117–2127. 10.1016/j.ejca.2011.05.007 21664811

[B16] Kudo M. Ueshima K. Ikeda M. Torimura T. Tanabe N. Aikata H. (2019). Randomised, Multicentre Prospective Trial of Transarterial Chemoembolisation (TACE) Plus Sorafenib as Compared with TACE Alone in Patients with Hepatocellular Carcinoma: TACTICS Trial. Gut 69, 1492–1501. 10.1136/gutjnl-2019-318934 31801872 PMC7398460

[B17] Lencioni R. de Baere T. Soulen M. C. Rilling W. S. Geschwind J.-F. H. (2016a). Lipiodol Transarterial Chemoembolization for Hepatocellular Carcinoma: A Systematic Review of Efficacy and Safety Data. Hepatology 64 (1), 106–116. 10.1002/hep.28453 26765068

[B18] Lencioni R. Llovet J. M. Han G. Tak W. Y. Yang J. Guglielmi A. (2016b). Sorafenib or Placebo Plus TACE with Doxorubicin-Eluting Beads for Intermediate Stage HCC: The SPACE Trial. J. Hepatol. 64 (5), 1090–1098. 10.1016/j.jhep.2016.01.012 26809111

[B19] Li X. Feng G. S. Zheng C. S. Zhuo C. K. Liu X. (2004). Expression of Plasma Vascular Endothelial Growth Factor in Patients with Hepatocellular Carcinoma and Effect of Transcatheter Arterial Chemoembolization Therapy on Plasma Vascular Endothelial Growth Factor Level. Wjg 10 (19), 2878–2882. 10.3748/wjg.v10.i19.2878 15334691 PMC4572123

[B20] Marrero J. A. Kudo M. Venook A. P. Ye S.-L. Bronowicki J.-P. Chen X.-P. (2016). Observational Registry of Sorafenib Use in Clinical Practice across Child-Pugh Subgroups: The GIDEON Study. J. Hepatol. 65 (6), 1140–1147. 10.1016/j.jhep.2016.07.020 27469901

[B21] Marrero J. A. Kulik L. M. Sirlin C. B. Zhu A. X. Finn R. S. Abecassis M. M. (2018). Diagnosis, Staging, and Management of Hepatocellular Carcinoma: 2018 Practice Guidance by the American Association for the Study of Liver Diseases. Hepatology 68 (2), 723–750. 10.1002/hep.29913 29624699

[B22] Meyer T. Fox R. Ma Y. T. Ross P. J. James M. W. Sturgess R. (2017). Sorafenib in Combination with Transarterial Chemoembolisation in Patients with Unresectable Hepatocellular Carcinoma (TACE 2): a Randomised Placebo-Controlled, Double-Blind, Phase 3 Trial. Lancet Gastroenterol. Hepatol. 2 (8), 565–575. 10.1016/S2468-1253(17)30156-5 28648803

[B23] Park J.-W. Kim Y. J. Kim D. Y. Bae S.-H. Paik S. W. Lee Y.-J. (2019). Sorafenib with or without Concurrent Transarterial Chemoembolization in Patients with Advanced Hepatocellular Carcinoma: The Phase III STAH Trial. J. Hepatol. 70 (4), 684–691. 10.1016/j.jhep.2018.11.029 30529387

[B24] Park J. W. Chen M. Colombo M. Roberts L. R. Schwartz M. Chen P. J. (2015). Global Patterns of Hepatocellular Carcinoma Management from Diagnosis to Death: the BRIDGE Study. Liver Int. 35 (9), 2155–2166. 10.1111/liv.12818 25752327 PMC4691343

[B25] Terzi E. Golfieri R. Piscaglia F. Galassi M. Dazzi A. Leoni S. (2012). Response Rate and Clinical Outcome of HCC after First and Repeated cTACE Performed "on Demand". J. Hepatol. 57 (6), 1258–1267. 10.1016/j.jhep.2012.07.025 22871502

[B26] Villanueva A. (2019). Hepatocellular Carcinoma. N. Engl. J. Med. 380 (15), 1450–1462. 10.1056/NEJMra1713263 30970190

[B27] Wang B. Xu H. Gao Z. Q. Ning H. F. Sun Y. Q. Cao G. W. (2008). Increased Expression of Vascular Endothelial Growth Factor in Hepatocellular Carcinoma after Transcatheter Arterial Chemoembolization. Acta Radiol. 49 (5), 523–529. 10.1080/02841850801958890 18568538

[B28] Wang Q. Xia D. Bai W. Wang E. Sun J. Huang M. (2019). Development of a Prognostic Score for Recommended TACE Candidates with Hepatocellular Carcinoma: A Multicentre Observational Study. J. Hepatol. 70 (5), 893–903. 10.1016/j.jhep.2019.01.013 30660709

[B29] Weinmann A. Koch S. Sprinzl M. Kloeckner R. Schulze-Bergkamen H. Düber C. (2015). Survival Analysis of Proposed BCLC-B Subgroups in Hepatocellular Carcinoma Patients. Liver Int. 35 (2), 591–600. 10.1111/liv.12696 25290314

[B30] Zhong B.-Y. Ni C.-F. Chen L. Zhu H.-D. Teng G.-J. (2017). Early Sorafenib-Related Biomarkers for Combination Treatment with Transarterial Chemoembolization and Sorafenib in Patients with Hepatocellular Carcinoma. Radiology 284 (2), 583–592. 10.1148/radiol.2017161975 28263701

